# Supplementary material for: Conditional chemoconnectomics (cCCTomics) as a strategy for efficient and conditional targeting of chemical transmission
Source: eLife. 2024 Apr 30;12:RP91927. doi: 10.7554/eLife.91927 (PMC11060718; doi:10.7554/eLife.91927)
Supplement: Figure 3—source data 3. [file elife-91927-fig3-data3.zip › Figure 3 source data3 part 1.zip!/Figure 3-Source data 3-part1/1081-1-CRISPResso_Report_8zosk6yj.zip!/CRISPResso_on_1081_2/9.Alleles_frequency_table_around_sgRNA_tttctggactacagtcccca.pdf]

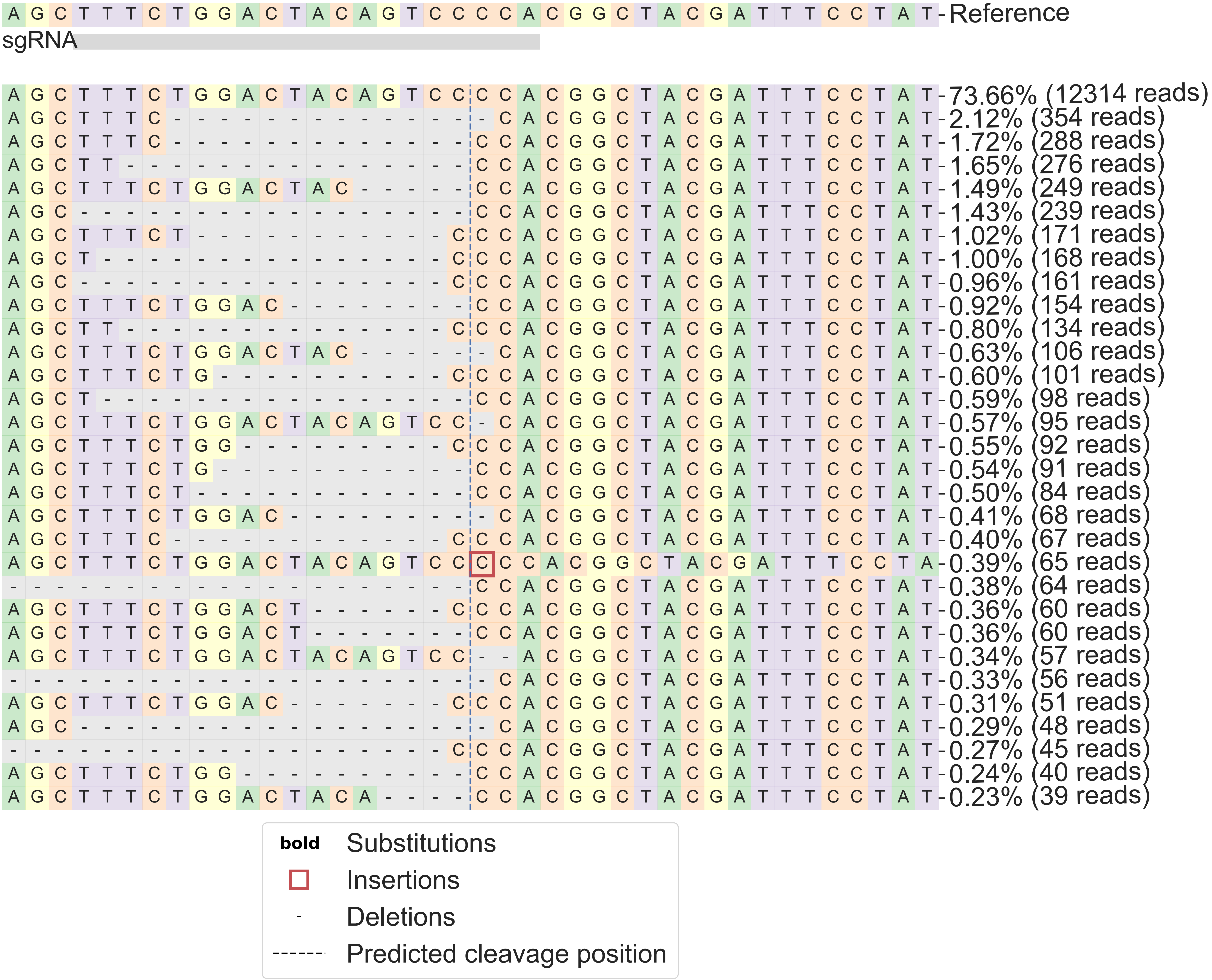

Reference
sgRNA
73.66% (12314 reads)
2.12% (354 reads)
1.72% (288 reads)
1.65% (276 reads)
1.49% (249 reads)
1.43% (239 reads)
1.02% (171 reads)
1.00% (168 reads)
0.96% (161 reads)
0.92% (154 reads)
0.80% (134 reads)
0.63% (106 reads)
0.60% (101 reads)
0.59% (98 reads)
0.57% (95 reads)
0.55% (92 reads)
0.54% (91 reads)
0.50% (84 reads)
0.41% (68 reads)
0.40% (67 reads)
0.39% (65 reads)
0.38% (64 reads)
0.36% (60 reads)
0.36% (60 reads)
0.34% (57 reads)
0.33% (56 reads)
0.31% (51 reads)
0.29% (48 reads)
0.27% (45 reads)
0.24% (40 reads)
0.23% (39 reads)
bold Substitutions
Insertions
Deletions
Predicted cleavage position
